# Pituitary Extract in Obstetrics

**Published:** 1914-01

**Authors:** 


Pituitary Extract in Obstetrics. Palmer Findley, M. D., Omaha, *Western Med. Rev.*, Nov., 1913. Pituitary extract finds its greatest field of usefulness in overcoming uterine inertia, and particularly in the second stage of labor.

The remedy is not to be used in normal labor, but should be restricted to cases in which the uterus is fagged, the cervix dilated or easily dilatable and the resistance of the soft parts and bony pelvis is not great. As a result of pituitrin short, feeble contractions are converted into long and strong contractions. The possibility of creating too strong and too prolonged contractions must be borne in mind, and it can never be foretold just what the effect of a given dose will be.

Polak speaks of a case in which the pains were so intense and so prolonged that morphine and chloroform were used to relax the uterus. A number of cases are on record in which the uterus was ruptured, the cervix badly lacerated and the child asphyxiated.

These unfortunate results can be largely averted by the judicious proportioning of the dosage and by eliminating all cases in which the cervix is not dilated or easily dilatable, and in which there is undue resistance in the vagina, the pelvic floor or the bony pelvis. Finally, by excluding cases in which a faulty presentation and position speaks for a difficult labor. Disregard of these contraindications in the administration of pituitrin may cause premature separation of the placenta, with uterine hemorrhage and death of the fetus, and the prolonged pressure of the tightly contracted uterus may injure the fetal head. To bring the presenting head within easier reach of the forceps a moderate dose of pituitrin may be given. It appears that the action of the drug is more positive in multipara than in primipara and at full term than in the earlier months of gestation. Its action is more prompt in the second than in the first or third stages of labor. It will not induce labor or abortion, nor will it aid in the expulsion of placental remains.

I have personally found it of very great assistance in hastening labor after the insertion of a hydrostatic bag. In this particular it becomes a very important adjunct to the usual means of inducing labor in placenta previa, eclampsia and such other conditions which demand the interruption of pregnancy. It is not a substitute for ergot in post partum hemorrhage, nor is it to replace morphine in uterine inertia where the mother is exhausted for want of rest. But it is a most valuable remedy in uterine inertia in the second stage of labor, in the absence of great resistance to the presenting part.

